# Exploration of the Molecular Mechanism of Polygonati Rhizoma in the Treatment of Osteoporosis Based on Network Pharmacology and Molecular Docking

**DOI:** 10.3389/fendo.2021.815891

**Published:** 2022-01-05

**Authors:** Jinlong Zhao, Fangzheng Lin, Guihong Liang, Yanhong Han, Nanjun Xu, Jianke Pan, Minghui Luo, Weiyi Yang, Lingfeng Zeng

**Affiliations:** ^1^ The Second Clinical Medical College of Guangzhou University of Chinese Medicine, Guangzhou, China; ^2^ The 2nd Affiliated Hospital of Guangzhou University of Chinese Medicine, Guangzhou, China

**Keywords:** Polygonati Rhizoma, potential therapeutic targets, signaling pathways, molecular mechanism, network pharmacology, molecular docking

## Abstract

**Objective:**

To explore the effective components and mechanism of Polygonati Rhizoma (PR) in the treatment of osteoporosis (OP) based on network pharmacology and molecular docking methods.

**Methods:**

The effective components and predicted targets of PR were obtained through the Traditional Chinese Medicine Systems Pharmacology and Analysis Platform (TCMSP) database. The disease database was used to screen the disease targets of OP. The obtained key targets were uploaded to the Search Tool for the Retrieval of Interacting Genes/Proteins (STRING) database for protein-protein interaction (PPI) network analysis. The Database for Annotation, Visualization, and Integrated Discovery (DAVID) was used for Gene Ontology (GO) and Kyoto Encyclopedia of Genes and Genomes (KEGG) pathway enrichment analyses of key targets. Analysis and docking verification of chemical effective drug components and key targets were performed with IGEMDOCK software.

**Results:**

A total of 12 chemically active components, 84 drug target proteins and 84 common targets related to drugs and OP were obtained. Key targets such as JUN, TP53, AKT1, ESR1, MAPK14, AR and CASP3 were identified through PPI network analysis. The results of enrichment analysis showed that the potential core drug components regulate the HIF-1 signaling pathway, PI3K-Akt signaling pathway, estrogen signaling pathway and other pathways by intervening in biological processes such as cell proliferation and apoptosis and estrogen response regulation, with an anti-OP pharmacological role. The results of molecular docking showed that the key targets in the regulatory network have high binding activity to related active components.

**Conclusions:**

PR may regulate OP by regulating core target genes, such as JUN, TP53, AKT1, ESR1, AR and CASP3, and acting on multiple key pathways, such as the HIF-1 signaling pathway, PI3K-Akt signaling pathway, and estrogen signaling pathway.

## Introduction

Osteoporosis (OP) is a systemic metabolic bone disease characterized by bone loss, bone tissue microstructure damage, bone pain and brittle fracture (1). The incidence of OP is closely related to age, and with the ageing of the population, OP has become an important factor affecting the quality of life of middle-aged individuals and elderly individuals. Research data show that 40% of postmenopausal women and 30% of men in the world will develop OP (2). In addition, OP may induce other diseases, which causes a heavy economic burden to the patient’s family and society (3). At present, most of the drugs for the treatment of OP are chemical drugs that regulate bone metabolism, but there are adverse reactions such as kidney injury and joint pain (4, 5). In recent years, many scholars have studied the efficacy of traditional Chinese medicine in the treatment of OP from the perspective of molecular biology and have made remarkable progress, further proving the positive role of traditional Chinese medicine in the treatment of OP (6, 7).

OP belongs to the category of “*Guwei*” in traditional Chinese medicine. It is recorded in *Su Wen* that “If the kidney *Qi* is hot, the waist and spine will not lift, the bone will wither and the marrow decreases, it will develop into *Guwei*”. The basic principles of traditional Chinese medicine treatment are tonifying the kidney and essence, promoting blood circulation and removing blood stasis (8, 9). The relevant molecular mechanism of traditional Chinese medicine in the treatment of OP has not been fully clarified, which restricts the further transformation of relevant traditional Chinese medicine theories into clinical practice. In the context of traditional Chinese medicine, it is believed that Polygonati Rhizoma (PR) has effects of tonifying the kidney, tonifying *Qi* and nourishing *Yin*, strengthening the spleen and moistening the lung, which is the theoretical basis for its anti-OP effects (10). Modern pharmacological studies have shown that PR has anti-OP, bone protection, neuroprotection and immune enhancement effects (10, 11). According to experimental studies, *Polygonatum sibiricum* polysaccharide, one of the main active components of PR, can effectively promote osteogenic differentiation of bone marrow stromal cells in mice and inhibit the formation of osteoclasts (12). *Polygonatum sibiricum* polysaccharide can also prevent and treat OP mainly by improving bone microstructure, reducing bone turnover, increasing OPG protein and reducing RANKL protein expression (13, 14). Network pharmacology systematically studies the potential targets and pharmacological effects of traditional Chinese medicine components on multiple genes, multiple targets and multiple ways, which provides a new idea for research on the modernization of traditional Chinese medicine (15). Molecular docking is a theoretical simulation method to predict a drug’s binding mode and affinity through the interaction between receptor and drug molecules (16). We aimed to use network pharmacological methods and molecular docking technology to preliminarily explore the potential targets and pathways of PR in the treatment of OP to provide a basis for further clarifying the molecular mechanism of PR in the treatment of OP.

## Materials and Methods

### Active Components and Action Targets of PR

The active components of PR were extracted from the Traditional Chinese Medicine Systems Pharmacology Database and Analysis Platform (TCMSP) (http://lsp.nwu.edu.cn/tcmsp.php) (15, 17). The compounds were screened according to oral bioavailability (OB) and drug likeness (DL). The screening conditions are OB ≥ 30% and DL ≥ 0.18 (18). The screened compounds are the core active components. The proteins corresponding to the above targets were transformed into species human genes through the UniProt database (https://www.UniProt.org/), and a database of PR compounds and their action target genes was constructed.

### Acquisition of OP Disease Targets

In the Comparative Toxicogenomics Database (CTD) (http://ctdbase.org/), Genecards (https://www.genecards.org/) and DisGeNET databases (http://www.disgenet.org/web/DisGeNET/menu/home), “osteoporosis” was used as the search term to collect disease targets related to OP. Finally, duplicates from the three databases were removed to obtain the final OP-related targets.

### Common Targets

The targets related to OP and the targets related to the active components of PR overlapped. The intersecting genes and Venn diagram of PR and OP targets were obtained through the online Venn map platform (http://bioinfogp.cnb.csic.es/tools/venny/index.html), and finally, OP-related targets of the drug active ingredients were obtained for subsequent analysis.

### Construction of the Compound-Target Network

Information on the active ingredients and common targets was imported into Cytoscape_v3.7.1 software to construct a compound-target interaction network to show the interaction relationships between active components and targets. The software calculates the network topology parameters, degree and betweenness centrality (BC) and screens the main candidate targets and monomer components.

### Protein-Protein Interaction Network

PPIs are the basis of cell function and play an important role in regulating physiological and pathological states. In the PPI network, network nodes represent proteins, network connecting lines represent PPIs, and node size, colour, connection length and thickness represent the topology parameters of the node network (19). To further understand the synergistic mechanism of the PR compound potential target and OP target at the protein level, the common target protein was passed through the Search Tool for the Retrieval of Interacting Genes/Proteins (STRING) 11.0 (https://string-db.org). The PPI relationship is obtained, and then the core gene is obtained according to the number of node connections. Among them, the species selection was “*Homo sapiens*”, and high confidence = 0.9 was set as the lowest interaction score.

### Gene Ontology and Kyoto Encyclopedia of Genes and Genomes Enrichment Analysis

We imported the obtained potential targets of PR for the treatment of OP (the common target obtained in 1.1.3) into the target gene name list through Database for Annotation, Visualization, and Integrated Discovery (DAVID) (https://david.ncifcrf.gov/home.jsp) and limited the species to human. GO enrichment analysis and KEGG pathway annotation analysis were performed on the potential targets of PR for the treatment of OP to screen the important signaling pathway of PR in OP treatment.

### Molecular Docking

The target proteins and ligand molecules were docked by IGEMDOCK docking software (http://gemdock.life.nctu.edu.tw/). The three-dimensional structure of the protein encoded by the top 15 core target genes from the Protein Data Bank (PDB) database (https://www.rcsb.org/) was downloaded as the protein receptor. The two-dimensional structure of the core active compound of PR was downloaded from the TCMSP database. The binding energy can be obtained by introducing them into IGEMDOCK. The binding ability of ligands and receptors is evaluated by the binding energy. If the binding energy is less than 0, it means that ligand and receptor can spontaneously bind, and the smaller the value, the higher the binding activity.

## Results

### Active Ingredients of PR

Thirty-eight chemical constituents of PR were retrieved from the TCMSP database. After screening with the parameters OB ≥ 30% and DL ≥ 0.18, 12 active components of PR were obtained. Since the corresponding targets were not selected for the three compounds (MOL003889, MOL009760 and MOL009766), subsequent analysis was not carried out. There were 84 corresponding targets of active components (excluding repeated targets). See **Table 1** for specific information on the screened active ingredients. A summary of putative targets of PR is provided in **Supplement Table 1**.

**Table 1:** Information on active ingredients of PR.

Molecule ID	Molecule Name	OB (%)	DL
MOL001792	DFV	32.76	0.18
MOL002714	baicalein	33.52	0.21
MOL002959	3’-Methoxydaidzein	48.57	0.24
MOL000358	beta-sitosterol	36.91	0.75
MOL000359	sitosterol	36.91	0.75
MOL003889	methylprotodioscin_qt	35.12	0.86
MOL004941	(2R)-7-hydroxy-2-(4-hydroxyphenyl)chroman-4-one	71.12	0.18
MOL000546	diosgenin	80.88	0.81
MOL006331	4’,5-Dihydroxyflavone	48.55	0.19
MOL009760	sibiricoside A_qt	35.26	0.86
MOL009763	(+)-Syringaresinol-O-beta-D-glucoside	43.35	0.77
MOL009766	zhonghualiaoine 1	34.72	0.78

### OP-Related Genes

With “osteoporosis” as the search term, 36180 related disease genes were retrieved from the CTD, 2916 were retrieved from the Genecards database, and 1098 were retrieved from the DisGeNET database. After merging the results from the three databases and removing duplicates, a total of 23530 genes related to OP were obtained. The relevant disease genes of the above three databases can be found in **Supplement Table 2**.

### Common Targets

**Figure 1:**
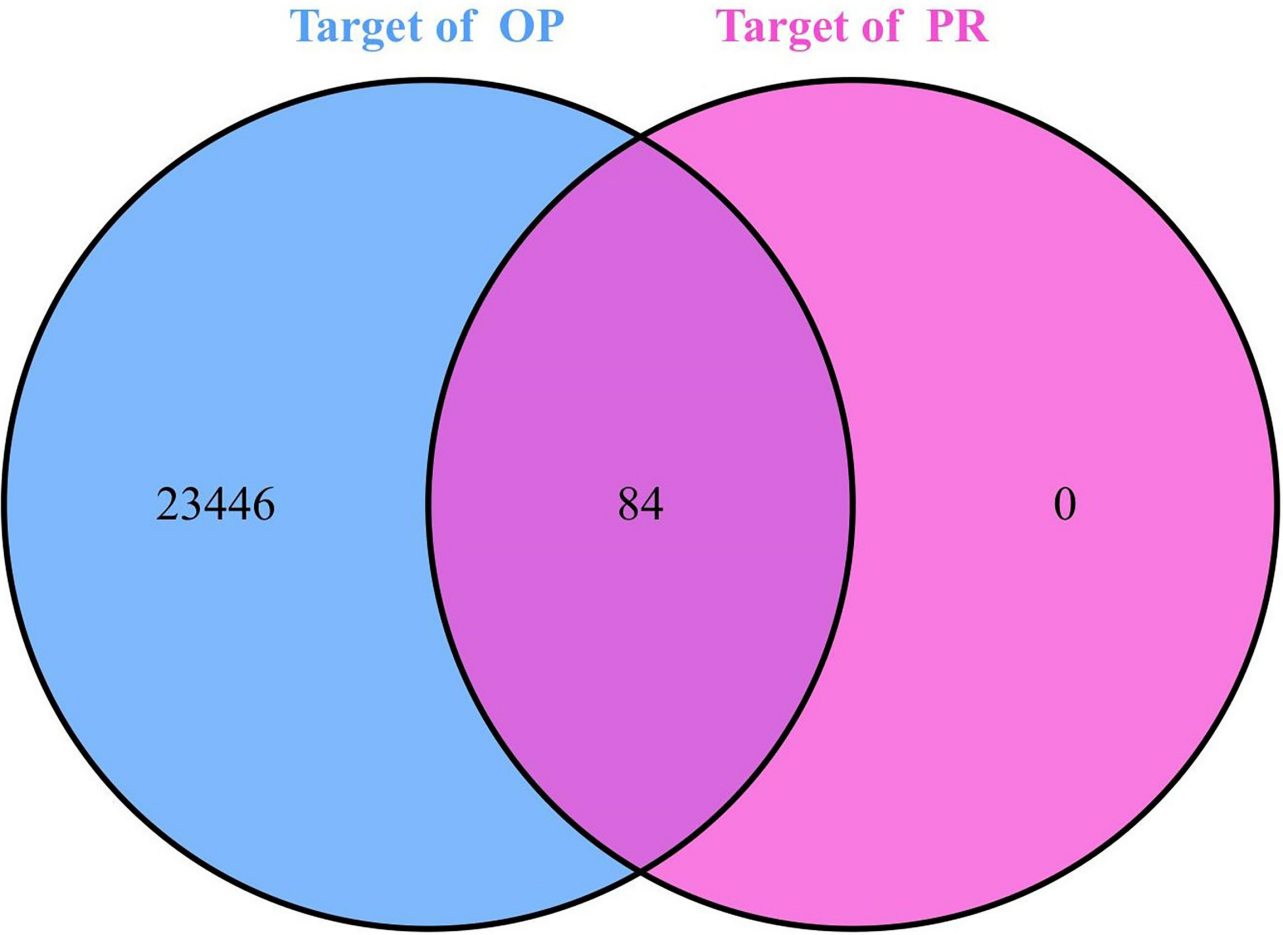
Venn diagram of OP targets and PR targets.

The results for drug active ingredient targets and disease targets were intersected, and 84 drug active ingredient targets related to OP were obtained for subsequent analysis (**Figure 1**).

### Construction and Analysis of the Compound-Target Network

**Figure 2:**
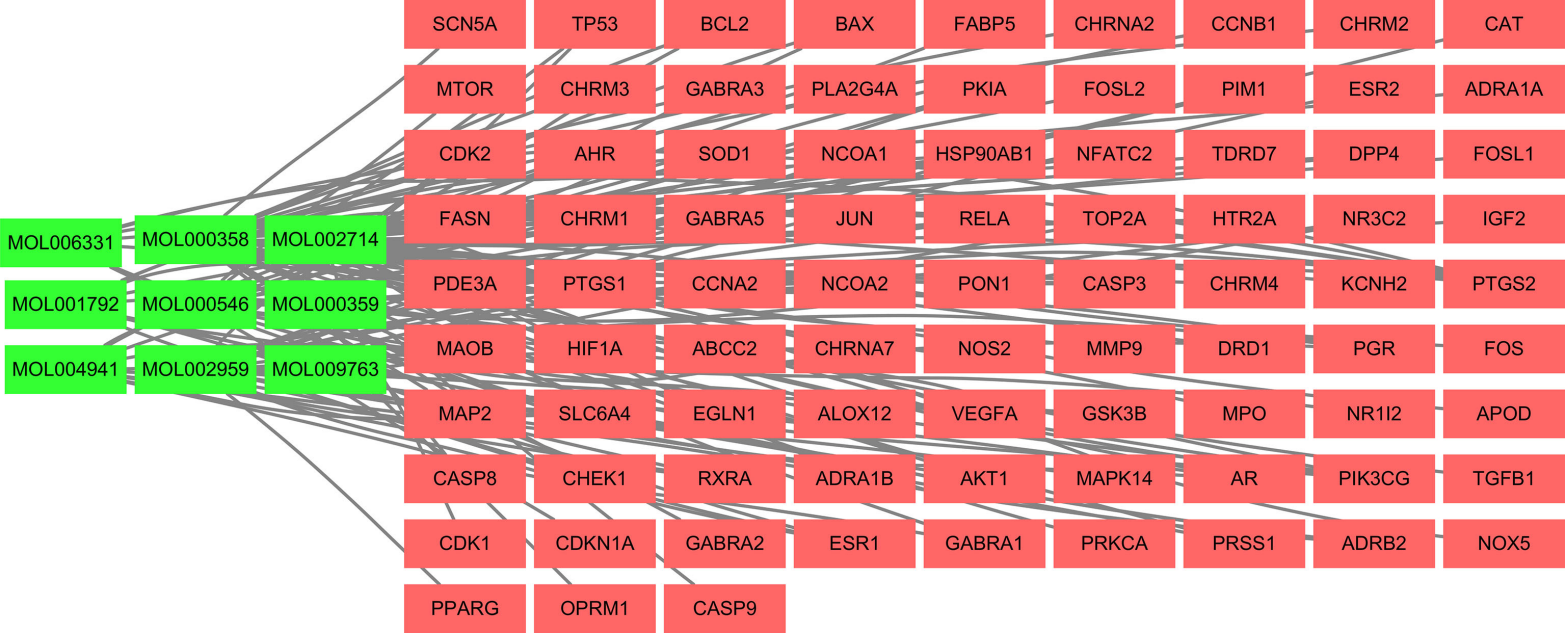
The gene target network of the effective components of PR.

Cytoscape 3.7.1 software was used to construct the active component target interaction network of 9 PR active monomers and 84 overlapping target genes. The green node represents the active component of PR, and the red node is the action target of the active component, with a total of 272 edges, representing the interaction between the target and the chemical component, reflecting the multicomponent and multitarget characteristics of PR (**Figure 2**). For network topology analysis, the network concentration was 0.367, the network density was 0.032, and the network heterogeneity was 1.891. The average degree value of nodes was 2.92, and there were 21 nodes with above average degree values. According to the topological properties of network nodes, such as degree value and BC, the core nodes were selected for analysis. The nodes with more connecting compounds or targets play a key role in the whole network and may be key compounds or targets. **Table 2** lists the 20 key nodes with above average value and their topological parameters in the compound-target network.

**Table 2:** Key node of the compound-target network and table of its topological features.

Node	Node type	Degree	BC	Node	Node type	Degree	BC
MOL000358	Monomer	36	0.49952593	PIK3CG	Target	5	0.03609123
MOL002714	Monomer	34	0.45840389	SLC6A4	Target	3	0.00550579
MOL002959	Monomer	17	0.21181913	RXRA	Target	3	0.00374525
MOL000546	Monomer	16	0.20289903	PGR	Target	3	0.0443633
MOL004941	Monomer	12	0.05237295	PDE3A	Target	3	0.02399951
MOL001792	Monomer	10	0.03656977	NCOA2	Target	3	0.03311465
PTGS2	Target	7	0.17893754	MOL000359	Monomer	3	0.00567139
MOL006331	Monomer	7	0.0153846	MAOB	Target	3	0.00062739
PTGS1	Target	6	0.077882	ESR1	Target	3	0.00374525
HSP90AB1	Target	6	0.077882	AR	Target	3	0.01422897

### PPI Network

**Figure 3:**
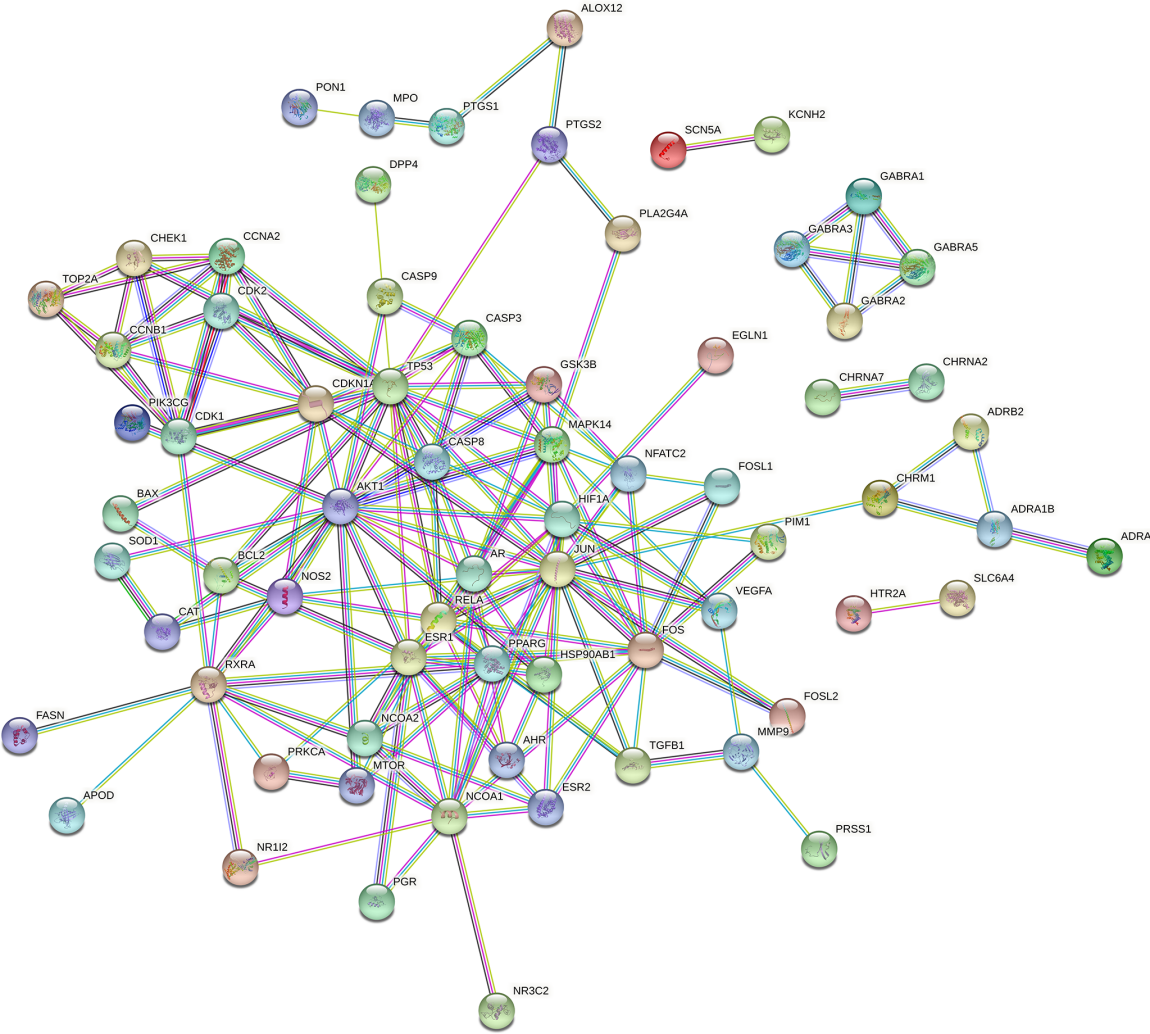
PPI network diagram of target proteins.

**Figure 4:**
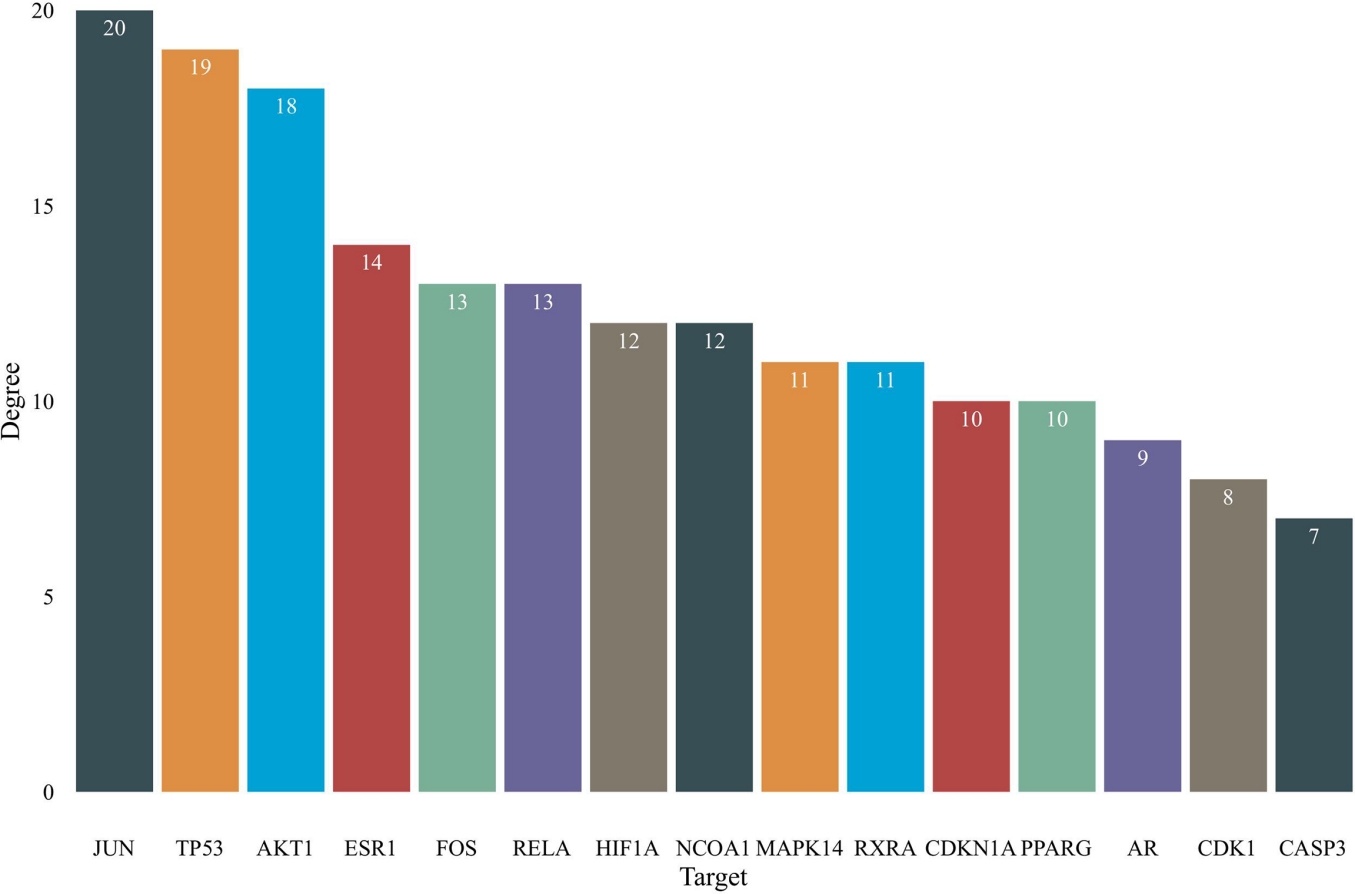
Core targets of PR in the treatment of OP (top 15).

The 84 intersecting genes obtained in “2.3” were imported into STRING 11.0 for analysis. The PPI results are shown in **Figure 3**. The PPI results included 70 nodes and 346 edges, in which the nodes represented proteins and each edge represented a PPI relationship, with an average degree of 4.94. **Figure 4** is a bar chart of the top 15 target genes, which are considered to be the key targets of PR for the treatment of OP.

### GO Enrichment Analysis

**Figure 5:**
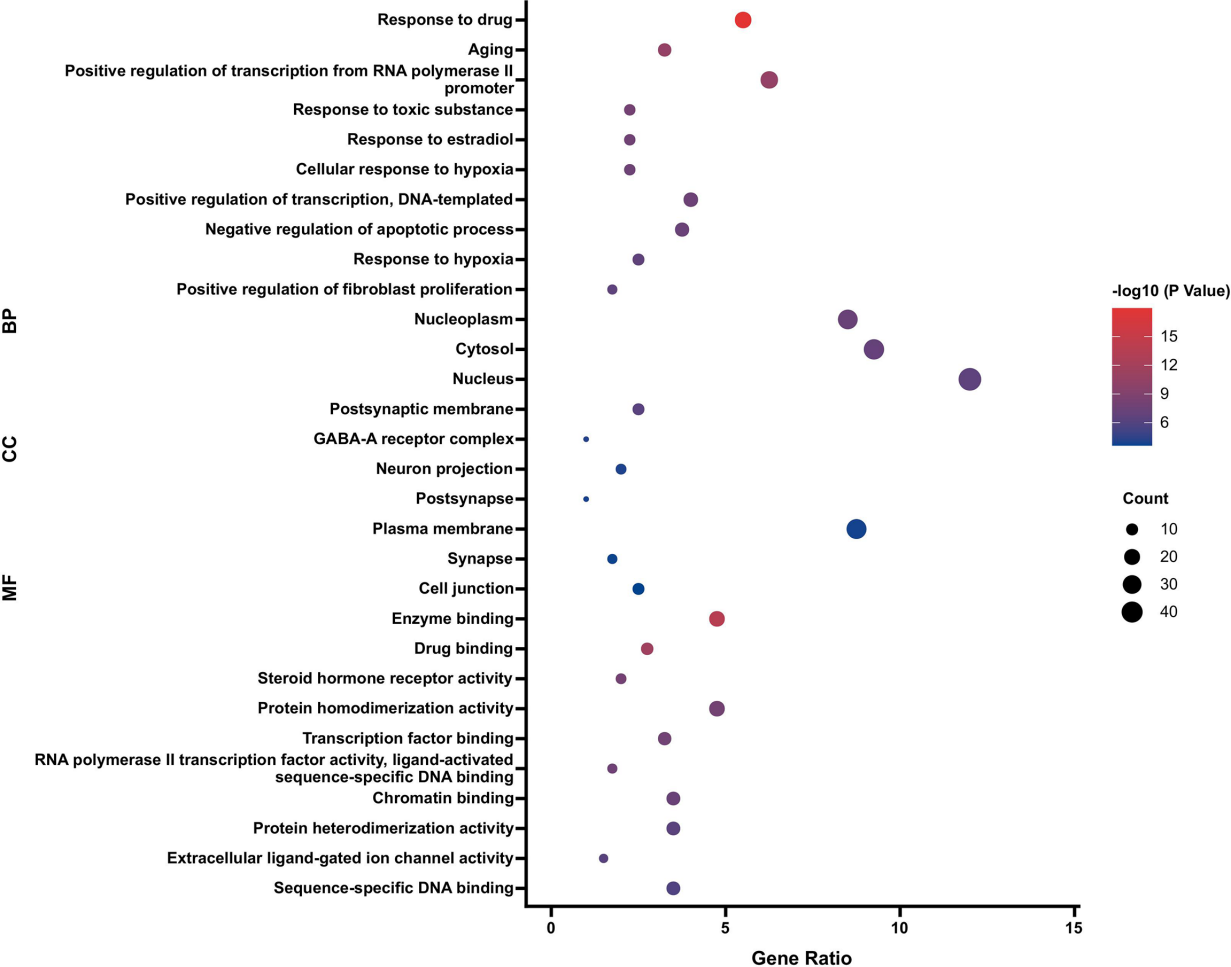
GO enrichment analysis of PR targets in the OP treatment (top 10).

By GO enrichment analysis of 84 target genes intersecting PR and OP, 881 GO items were obtained, including 356 biological process (BP), 46 cellular component (CC) and 79 molecular function (MF) terms. According to the *p* value and the number of enriched genes, the top 10 enriched BPs, CCs and MFs were selected for visualization, and a bubble diagram was generated (**Figure 5**). The BPs were mainly related to the response to drug, positive regulation of transcription from RNA polymerase II promoter, response to estradiol, cellular response to hypoxia, etc.; the CCs were mainly related to the nucleoplasm, cytosol, nucleus and postsynaptic membrane; and the MFs were mostly related to enzyme binding, drug binding and steroid hormone receptor activity.

### KEGG Pathway Enrichment Analysis

**Figure 6:**
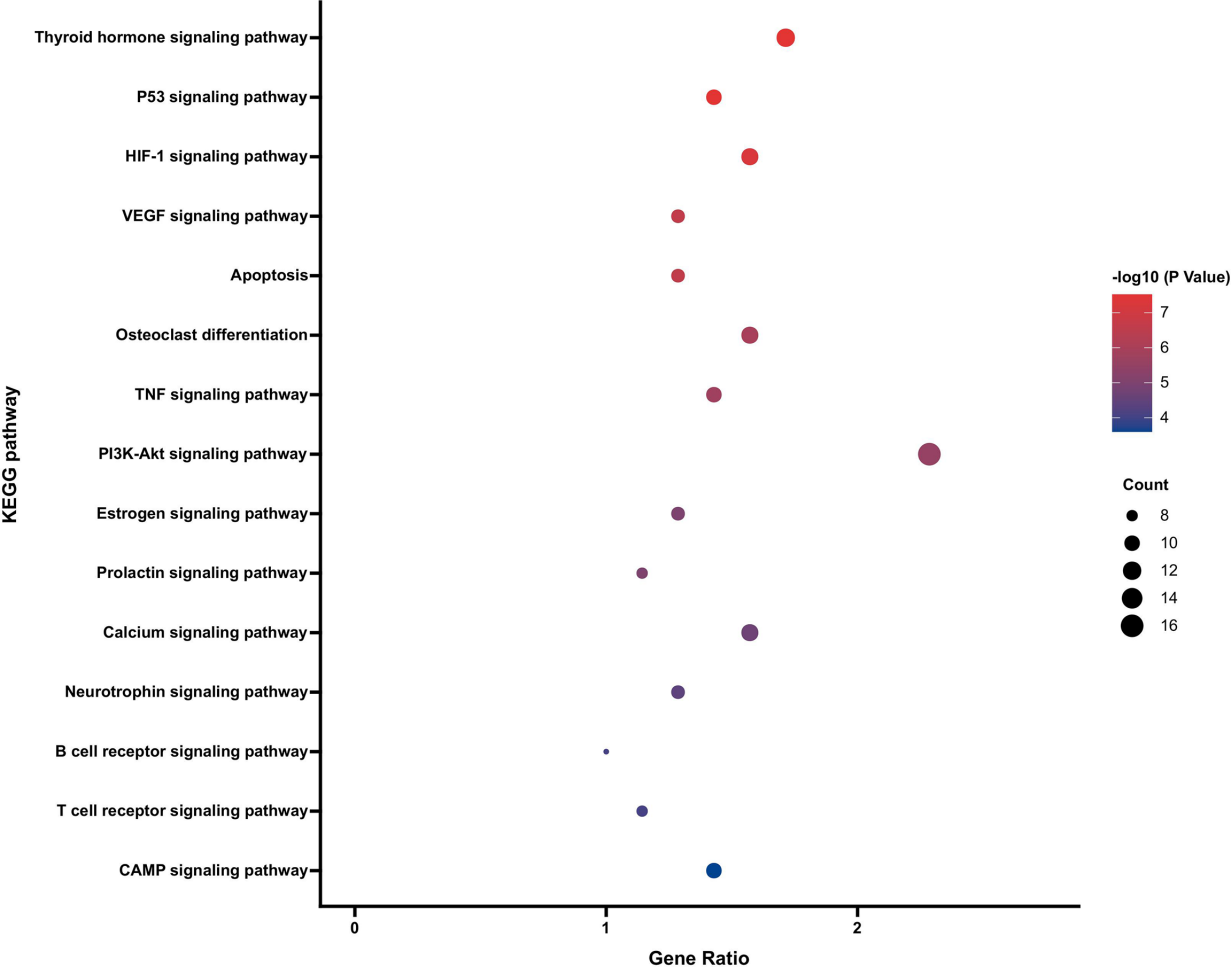
KEGG pathway enrichment analysis of PR targets in the treatment of OP (top 15).

KEGG analysis of the intersecting target genes of PR and OP showed that the key target genes were enriched in 94 biological signaling pathways. Using the Benjamin correction method, the top 15 noncancer disease pathways were analysed after ranking the *p* value, and the KEGG functional enrichment bubble diagram was drawn (**Figure 6**). The main biological pathways involved included the p53 signaling pathway, HIF-1 signaling pathway, VEGF signaling pathway, TNF signaling pathway, estrogen signaling pathway and other signaling pathways.

### Molecular Docking Results

**Figure 7:**
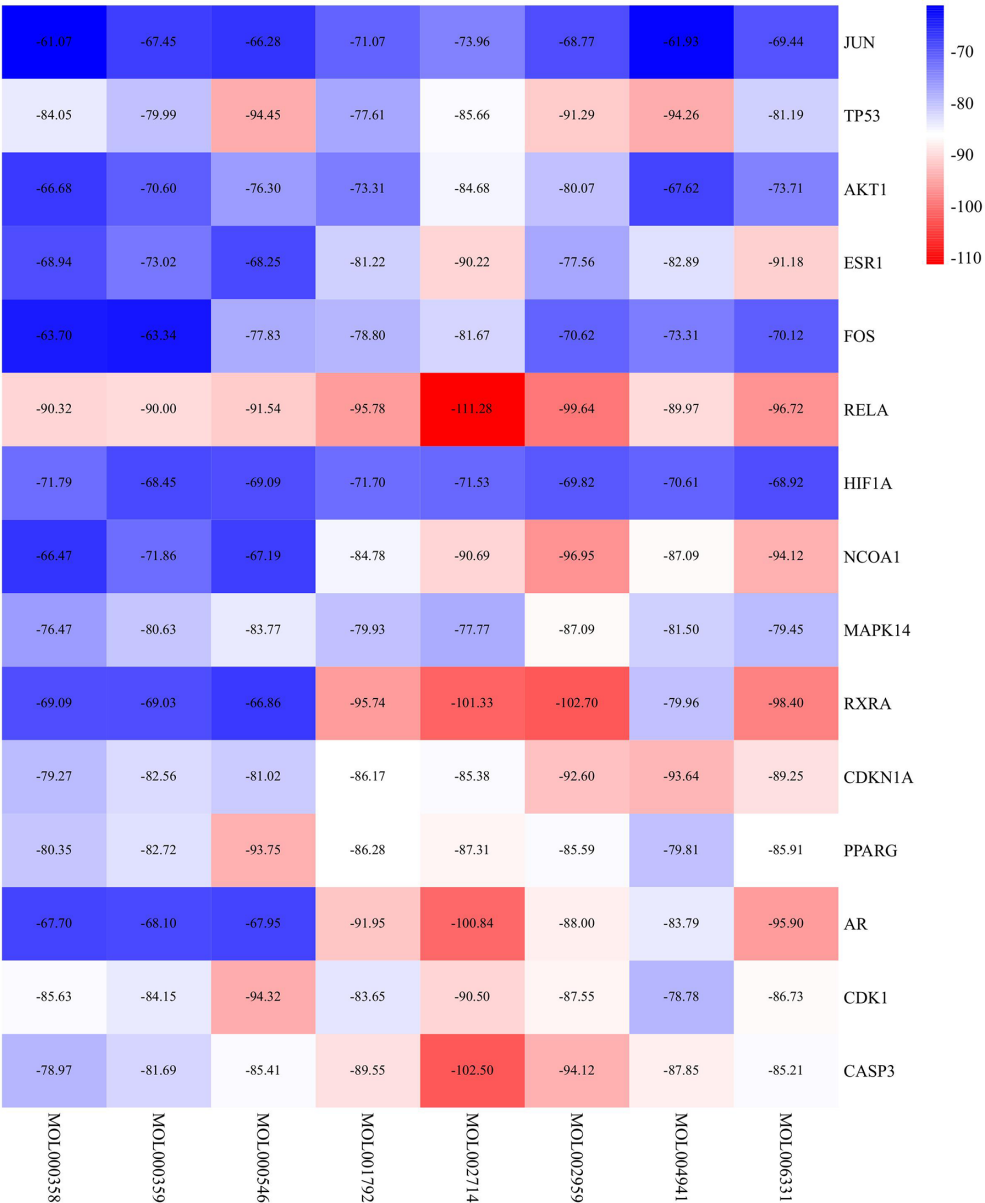
Molecular docking heatmap of the main compounds and key targets (Kcal/mol).

The key targets in the PPI network were selected, and the relevant components were searched reversely according to the PPI network for molecular docking. The lower the binding energy between ligand and receptor was, the more stable the binding conformation and the greater the possibility of interaction. The molecular docking results showed that the binding energy between the effective chemical active ingredient and the key target protein was less than -50 kcal/mol (**Figure 7**), suggesting that the binding activity between the effective core ingredient and the core target protein is stable.

## Discussion

With the acceleration of the ageing process worldwide, OP has become the third most common chronic disease, and it is a frequently occurring disease (20). The treatments for OP in modern medicine mainly include calcium, vitamin supplements and estrogen replacement therapy. However, the long-term use of estrogen can increase the incidence of breast cancer and coronary heart disease, and other chemical synthetic drugs used in the clinic have noneligible adverse reactions (1, 21). In the context of traditional Chinese medicine, deficiency of kidney essence is believed to be the main pathogenesis of OP. PR has the effect of tonifying the kidney, and modern pharmacological studies indicate that PR has an anti-OP effect, but its mechanism is still unclear. Using network pharmacology and molecular docking technology, this study clarified the molecular mechanism of PR in the treatment of OP from a micro-level perspective to provide a theoretical basis for the treatment of OP with traditional Chinese medicine.

The study shows that PR may act on key targets such as JUN, TP53, AKT1, ESR1, MAPK14, AR and CASP3 through a variety of chemical components, such as baicalein, beta-sitosterol, diosgenin and 3’-methoxydaidzein, and regulate biological processes, such as estrogen regulatory response, cell antioxidant and antiaging signaling, cell proliferation and apoptosis, to further exert its multicomponent, multitarget and multinetwork anti-OP effect by regulating the p53 signaling pathway, HIF-1 signaling pathway, TNF signaling pathway, PI3K Akt signaling pathway and estrogen signaling pathway. A previous study suggested that *Polygonatum* polysaccharide may increase the contents of GPR48, BMP-2 and bone metabolic factors in bone tissue, improving the biomechanical properties and bone mineral density of osteoporotic fracture rats and delaying OP progression (11). Considering these findings and our research results, further experimental and clinical studies should explore the effect of PR on bone metabolism-related factors *via* intervention of JUN, TP53, AKT1, ESR1 and other proteins, which is very important for assessing PR in future drug development.

The active components with high degree values in the component-target network included beta-sitosterol, baicalein and other chemically active components. Beta-sitosterol widely exists in a variety of traditional Chinese medicine components and has physiological effects such as anti-inflammatory, antioxidant and anti-androgen activities (22). Studies have shown that beta-sitosterol can inhibit cell proliferation in transplanted tumours in mice by inhibiting the expression of IL-6, TNF and VEGF (23). TNF-α and IL-6 can induce RANKL expression and promote osteoclast differentiation. Therefore, beta-sitosterol may have potential pharmacological effects on inhibiting osteoclast activity (24). The GO BP enrichment results reflect that the treatment process is highly correlated with the regulatory response of estrogen, cell antioxidant and anti-ageing processes, cell proliferation and apoptosis. The estrogen level is closely related to the activities of osteoblasts and osteoclasts. In the estradiol response, when estrogen is insufficient, RANKL is activated, inhibits osteoclast apoptosis and accelerates the osteoclast process. Osteoblasts can increase their activity and enhance the process of osteogenesis by combining with estrogen. The key components of PR and the biological process of pharmacological action are strongly related to the dysregulation of bone remodeling in OP. The molecular docking results showed that the binding energy of potential core active components and key targets of PR were less than -50 kcal/mol, suggesting that the potential active components of PR have good binding activity with key targets, which indicates that they may be potential active components and targets for the treatment of OP.

The PPI and KEGG pathway enrichment results suggest that the effective components of PR mainly act on JUN, TP53, AKT1, ESR1, MAPK14, AR, CASP3 and other targets to play an anti-OP role, and these targets are mainly mapped to key pathways such as the HIF-1 signaling pathway, PI3K-Akt signaling pathway and estrogen signaling pathway. The HIF-1 signaling pathway is a classic multifunctional signaling pathway that plays an important role in bone formation and absorption. It can regulate the regeneration process of bone and blood vessels through osteogenic and angiogenic coupling (25, 26). In a hypoxic cell environment, it can widely participate in biological processes such as energy metabolism, angiogenesis and the cell cycle and can effectively strengthen the osteogenic and angiogenic differentiation ability of bone marrow mesenchymal stem cells (27). The PI3K-Akt signaling pathway mainly regulates cell proliferation, differentiation and apoptosis *in vivo*. After PI3K is activated, it recruits the downstream signaling molecule AKT, promotes mTOR activation to affect osteoblast differentiation and inhibit apoptosis, which has important guiding significance for the treatment of OP (28, 29). Estrogen can not only participate in the physiological processes of osteoclasts and osteoblasts but can also maintain the dynamic and stable balance of the abilities of the two cells and then affect the proliferation and differentiation of mesenchymal stem cells in the direction of osteogenesis (30, 31). Estrogen deficiency can cause in imbalance in osteoclast levels and osteogenesis, leading to OP (31). The PPI results in this study suggest that estrogen-related targets such as ESR1 and AR are the key anti-OP targets of PR. Therefore, the estrogen signaling pathway may be an important way for PR to exert its anti-OP effect.

This study also has some limitations. First, research methods based on network pharmacology have the disadvantages of being unable to predict up-regulation and down-regulation of targets, which is not conducive to an accurate understanding of the mechanism of chemical components acting on disease targets. Second, due to the limitation of screening conditions, only the main compounds in PR were analysed, restricting the results to a certain extent. Third, although a large number of targets and pathways can be screened through network pharmacology technology, the findings need to be confirmed through basic research and clinical trials, which is the focus of our next study.

## Conclusion

Based on the methodology of network pharmacology, through the evaluation of pharmacokinetic parameters of PR and the enrichment analysis of biological functional pathways, this study further verified that PR has multicomponent, multitarget and multipathway pharmacological characteristics in the treatment of OP. The interaction mode between chemically active components and OP disease targets was preliminarily verified by molecular docking, which provided an experimental basis for further biological experiments *in vivo* and *in vitro*.

## Data Availability Statement

The data sets presented in this study can be found in online repositories. The names of the repository/repositories and accession number(s) can be found in the article/**Supplementary Material**.

## Author Contributions

WY and LZ conceived this project. JZ, FL, and GL designed the study. ML and YH searched the literature data. NX and YH extracted the data. JZ, WY, and GL, ML, and JP drafted the manuscript. All authors contributed to the article and approved the submitted version.

## Funding

This work was supported by the National Natural Science Foundation of China (82004383, 81974574), the National key research and development program (2021YFC1712804), the Science and Technology Research Project of Guangdong Provincial Hospital of Chinese Medicine (YN2019ML08), China Postdoctoral Science Foundation (No. 2018M633036) and Medical Scientific Research Foundation of Guangdong Province (No. B2019091).

## Conflict of Interest

The authors declare that the research was conducted in the absence of any commercial or financial relationships that could be construed as a potential conflict of interest.

## Publisher’s Note

All claims expressed in this article are solely those of the authors and do not necessarily represent those of their affiliated organizations, or those of the publisher, the editors and the reviewers. Any product that may be evaluated in this article, or claim that may be made by its manufacturer, is not guaranteed or endorsed by the publisher.
